# Development and two-city validation of a Temperature-Sensitive Air Quality Health Index incorporating synergistic effects of air pollution and thermal stress

**DOI:** 10.3389/fpubh.2026.1806127

**Published:** 2026-08-26

**Authors:** Chuchu Luo, Haoxian He, Yiting Li, Nengzhou Chen, Chufang Wang, Yu Sun, Guang Hao, Xi Fu

**Affiliations:** 1 Guangdong-Hong Kong-Macao Joint Laboratory for Contaminants Exposure and Health, Guangdong Provincial Key Laboratory of Pharmaceutical Preparations Research and Evaluation and Guangdong Provincial Engineering Center of Topical Precise Drug Delivery System, School of Public Health, Guangdong Pharmaceutical University, Guangzhou, China; 2 Department of Urology, Liwan Central Hospital of Guangzhou, Guangzhou, China; 3 State Key Laboratory of Swine and Poultry Breeding Industry, Guangdong Provincial Key Laboratory for the Development Biology and Environmental Adaptation of Agricultural Organisms, College of Life Sciences, South China Agricultural University, Guangzhou, Guangdong, China

**Keywords:** climate change, extreme temperature, mortality, multi-pollutant model, temperature-sensitive

## Abstract

**Background:**

Ambient air pollution and non-optimal temperatures are two major environmental risk factors that interact to amplify health risks. Current Air Quality Health Indices (AQHI) are vital public health tools for warning against air pollution risk, but fail to account for the synergistic health effects of air pollution and non-optimal temperature.

**Objective:**

This study aimed to develop and validate a generalizable methodological framework for a Temperature-Sensitive Air Quality Health Index (TS-AQHI) that captures the synergistic effects of air pollution and temperature stress.

**Methods:**

We first constructed the TS-AQHI using Weighted Quantile Sum (WQS) regression as a preprocessing step to estimate pollutant weights, which were then entered as a weighted index into a primary Generalized Additive Model (GAM) framework incorporating temperature, humidity, and multiplicative interaction terms with two-day-lagged exposures. We then applied this framework to daily mortality, air pollution, and meteorological data from Ningbo (2014–2017) and London (2014–2017). Model validation was performed through quartile-based risk categorization using Poisson regression, time-series alignment with mortality, seasonal stratification, and temperature-threshold comparisons, with the conventional AQHI serving as the benchmark.

**Results:**

TS-AQHI significantly outperformed the conventional AQHI. In Ningbo, TS-AQHI showed 52.7% higher excess mortality risk (ER) for high-risk groups (ER: 22.9, 95% CI: 21.1–24.7 vs. 15.0, 95% CI: 13.4–16.7). In London, TS-AQHI demonstrated substantially greater predictive power for high-risk groups (ER: 22.2, 95% CI: 20.7–23.8 vs. 7.7, 95% CI: 6.3–9.0). Stratified analyses found that TS-AQHI consistently explained a higher proportion of mortality variance across all subgroups, particularly among older adults (≥65 years; *R^2^* = 25.1% vs. 20.5% for AQHI) and females (20.3% vs. 16.0%).

**Conclusion:**

This study presents a novel health risk index that integrates the synergistic effects of temperature and multi-pollutant exposure. The TS-AQHI provides a more robust and sensitive tool for public health agencies, enabling more accurate and timely warnings against the increasing threat of concurrent environmental exposures.

## 1 Introduction

Ambient air pollution and non-optimal temperatures are two major environmental risk factors that interact to amplify health risks. GBD 2021 estimates air pollution caused ~8.1 million premature deaths (58% from PM_2.5_) (1, 2), with long-term NO_2_ and short-term PM/O_3_ exposures associated with mortality and a 0.59% mortality increase per 10 μg/m^3^ SO_2_ (3–5). In the context of climate change, non-optimal ambient temperatures also impose a large health burden (6–9). Crucially, temperature and air pollution interact to amplify health risks (10). A large-scale study covering 620 cities in 36 countries found that high temperatures significantly enhance the mortality effects of PM_10_ and O_3_ (11). Similar synergistic interactions have been reported for cold temperatures and particulate matter in multi-city studies (12). This synergy implies that health risks on days with combined high pollution and extreme temperatures are substantially greater than the sum of their individual effects (13, 14). Thus, a health risk assessment tool that accounts for the synergistic interaction between multi-pollutant exposure and temperature is urgently needed, enabling timely predictions based on readily available air quality data.

Currently, to communicate the daily health risks, public health agencies have adopted indices such as the US EPA’s Air Quality Index (AQI) (15). However, the AQI relies on single-pollutant thresholds and does not capture the cumulative health effects of multi-pollutant mixtures (16). To address this limitation, the Air Quality Health Index (AQHI) addresses this by integrating excess mortality risks from multiple pollutants (e.g., NO_2_, SO_2_, PM) within a no-threshold, additive concentration-response framework (15, 17). The AQHI has been widely adopted because it better predicts hospitalizations for asthma, stroke, and other conditions compared to the AQI (18–20). Subsequent refinements have focused on statistical methods, including Bayesian weighting and quantile regression, to improve the index’s predictive performance (21, 22). Nevertheless, a critical limitation remains across all existing AQHI formulations: the complete omission of temperature extremes (23–25). A few composite indices that explicitly incorporate both air pollution and temperature have been proposed (26). In Monterrey, Mexico, a combined Air Quality and Heat Index (HI) was developed by testing additive or multiplicative combinations of AQI and HI, but it relied on the single-pollutant AQI and a short study period (25). In Canada, an AQHI-Plus was introduced to adjust for temperature during cold seasons, mainly to improve PM_2.5_ sensitivity during wildfire events, where temperature was treated as a covariate for adjustment rather than as an integral component with a formal interaction term (27). In summary, existing health risk indices do not incorporate temperature as an independent component nor account for its synergistic amplification with air pollution, which leads to systematic underestimation of health risks during cold or hot extremes in practice. Thus, a temperature-sensitive index that captures the synergistic amplification between temperature and air pollution is urgently needed for more accurate public health warnings.

The aim of our study is to propose and validate a Temperature-Sensitive Air Quality Health Index (TS-AQHI) that integrates the temperature into the multi-pollutant AQHI framework to capture the synergistic health effects of thermal stress and air pollution. This framework is designed to be applicable to other cities with similar data availability. To test its generalizability, we independently validated it in two climatically distinct cities: Ningbo, China (subtropical, with hot and humid summers) and London, UK (temperate, with cold and wet winters). In each city, we applied the same framework to full-year local data, constructing city-specific TS-AQHIs. We hypothesized that TS-AQHI will significantly outperform the conventional AQHI in mortality prediction, especially on extreme temperature days, thereby providing a more robust tool for public health protection under climate change.

## 2 Materials and methods

### 2.1 Study design and data sources

This study followed a three-phase design: (1) both AQHI and TS-AQHI were constructed from six air pollutants using a WQS-GAM pipeline, with TS-AQHI additionally incorporating temperature-pollution interactions, whereas AQHI relied solely on pollutant mixtures; (2) the identical framework was independently applied to both cities and evaluated against observed daily mortality counts to quantify differences in risk prediction performance; and (3) exploratory subgroup analyses restricted to Ningbo, which uniquely contained stratified mortality data.

Ningbo, China: daily data from 1 January 2014 to 31 December 2017 were obtained from the National Science and Technology Basic Resources Survey Program (Project: “Scientific Survey of Meteorological Sensitive Diseases in China’s Regional Population,” No. 2017FY101200). The dataset included daily counts of all-cause mortality (total and stratified by age and gender), daily concentrations of six air pollutants (NO_2_, SO_2_, CO, O_3_, PM_10_, and PM_2.5_), and daily meteorological variables (mean temperature, maximum temperature, and relative humidity, with units of °C and %, respectively). All data were aggregated at the city level from multiple monitoring stations across Ningbo. The temporal resolution is daily.

London, United Kingdom: daily data from 1 January 2014 to 30 June 2017 were obtained from three sources. Mortality data comprised all certified deaths of adults (≥18 years) registered in London, extracted from the Office for National Statistics (ONS) mortality registry for England and Wales (28). Air pollution and meteorological data were retrieved from three Automatic Urban and Rural Network (AURN) monitoring stations in London: London Bloomsbury, London N. Kensington, and Camden Kerbside, City of London (Supplementary Figure S1) (29, 30). The same six air pollutants and meteorological variables (mean temperature, maximum temperature, and relative humidity) were collected. City-level daily values were calculated as the arithmetic mean of the three stations. The temporal resolution is daily, and the spatial resolution is city-level. A summary of data sources, variables, and spatiotemporal resolution for both cities is provided in Supplementary Table S1.

### 2.2 Statistical analysis

The statistical analysis in each city proceeded in three steps. Step 1 (index construction) used WQS regression to derive city-specific pollutant weights and GAM to establish the exposure-response functions for both AQHI and TS-AQHI. Step 2 (model validation) compared the performance of the two indices through quartile-based risk categorization, time-series alignment, seasonal stratification, and temperature-threshold comparisons in both cities. Step 3 (exploratory subgroup analyses) was restricted to Ningbo, where stratified mortality data were available, to examine effect modification by age and sex. All primary models were fitted using GAM with a quasi-Poisson link function to account for overdispersion in daily mortality counts.

#### 2.2.1 Step 1-index construction: multi-pollutant AQHI

In this study, WQS regression was employed exclusively as a preprocessing step to derive city-specific pollutant weights. It constructs a weighted sum of the quantiled exposures, where the weights are empirically estimated to reflect each pollutant’s relative contribution to the overall mixture effect. In each validation city, we applied WQS to analyze six air pollutants using that city’s own daily mortality and pollutant data, resulting in the generation of a city-specific weighted index representing the mixture. The chemical-specific weights are interpreted as relative importance levels using similarly scaled variables (31–33). The resulting WQS-weighted pollutant index served as the sole exposure term in the primary analytical model, a GAM, that additionally adjusted for temporal trends and day-of-week effects, without including temperature or humidity as covariates (34). Detailed formulas are provided in Supplementary Equations S1, S2.

The AQHI was scored on a 1–10^+^ scale and classified into four health risk categories according to the standardized Canadian grading system (35): Low Risk (1–3), Moderate Risk (4–6), High Risk (7–10), and Very High Risk (10^+^).

#### 2.2.2 Step 1-index construction: TS-AQHI with temperature interaction

TS-AQHI extended the AQHI framework by incorporating temperature, humidity, and their multiplicative interaction within the same GAM structure. The WQS-derived weighted pollutant index from Section 2.2.1 was retained unchanged. We additionally constructed a temperature (tempdev represents the deviation from the temperature associated with the lowest mortality risk in each city.) and humidity (50%) deviation variable (7, 36). To capture the synergistic health effects of combined air pollution and bidirectional thermal stress, which is a key limitation of the conventional AQHI, we constructed the TS-AQHI by air pollution, temperature, humidity, and their interactions within a GAM framework, lagged by 2 days (37). We compared alternative interaction specifications and selected the optimal interaction model based on the lowest Akaike Information Criterion (AIC) and Bayesian Information Criterion (BIC) values (Supplementary Table S2) (38, 39). The Excess Risk (ER) estimated from this interactive model was then used to construct the TS-AQHI (Supplementary Equation S3).


$$\begin{array}{c} \ln(E[Y_{t}])=\alpha +\beta_{i}\mathrm{mix}\_\text{weight}\_\text{airpollutio}n_{t-2}+\beta_{i}{\text{tempdev}}_{t-2}\\ +\beta_{i}\text{RHde}v_{t-2}+\beta_{i}(\text{RHde}v_{t-2}*{\text{tempdev}}_{t-2})+\mathrm{ns}(\text{time},df_{T})\\ +\mathrm{ns}(\mathrm{dow},df_{T}) \end{array}$$

Where:

-
$Y_{t}$ denotes the all-cause death on day t.
-
$\mathrm{mix}\_\text{weight}\_\text{airpollutio}n_{t-2}$ refers to a weighted air pollutant index, lagged by 2 days.
-
$\text{RHde}v_{t-2}$ is the deviation of mean relative humidity from 50%, lagged by 2 days.
-
${\text{tempdev}}_{t-2}$ is the deviation of reference temperature (22.8 °C for Ningbo and 19.5 °C for London), lagged by 2 days.
-
$\beta_{i}(\text{RHde}v_{t-2}*{\text{tempdev}}_{t-2})$ is the multiplicative interaction term between the humidity deviation and the temperature deviation on day t.
-
$\;\beta_{i}$ represents the coefficient for the effect.
-
$\mathrm{ns}(\text{time},df_{T})$ is a natural cubic spline of time with df_T_ degrees of freedom.
-
$\mathrm{ns}(\mathrm{dow},df_{T})$ is a natural cubic spline of day of the week with df_T_ degrees of freedom.
-
α is the intercept.

**Figure 1:**
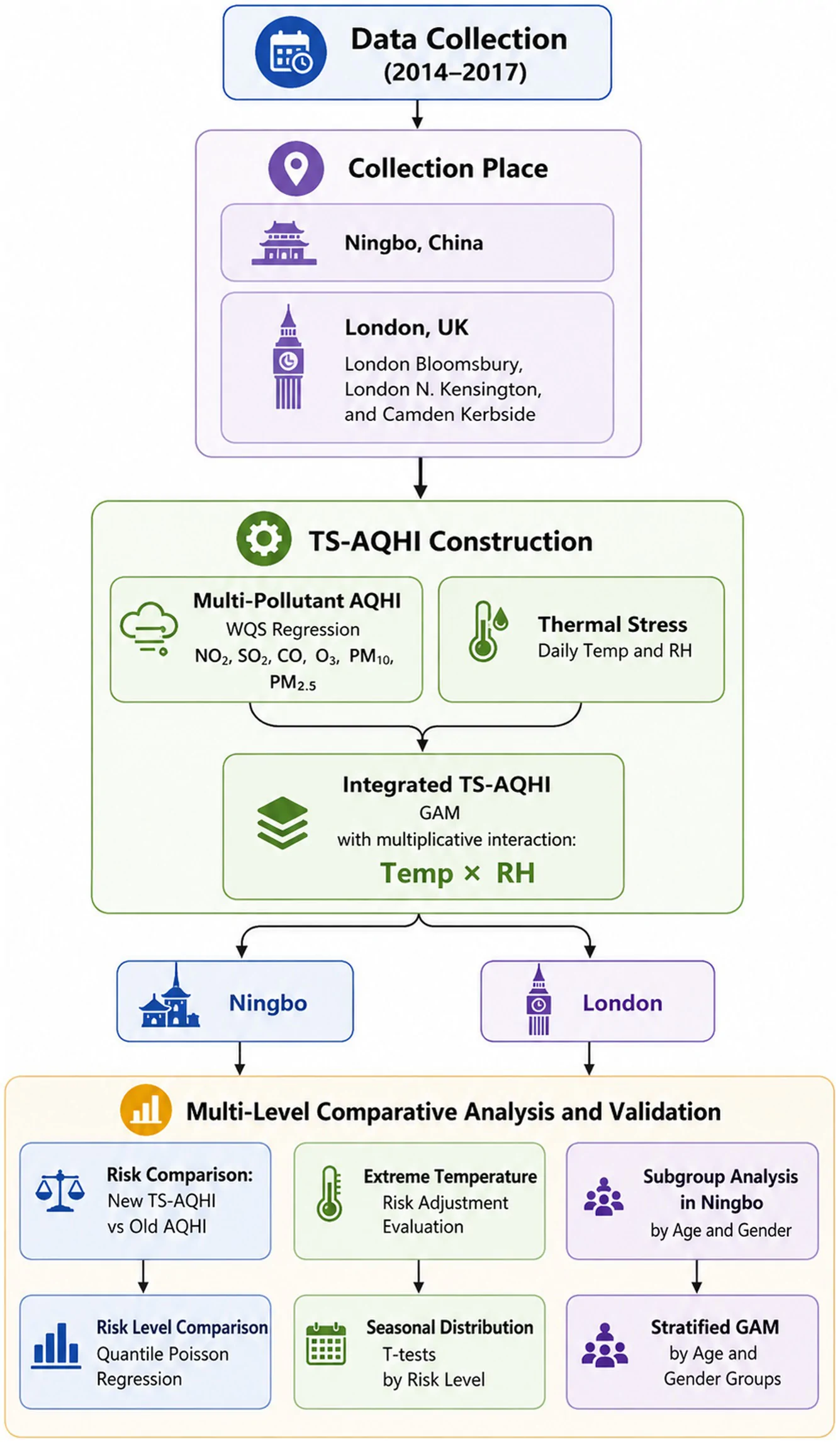
Flowchart illustrating the development and validation of the Temperature-Sensitive Air Quality Health Index (TS-AQHI).

This three-step procedure is applied independently to Ningbo and London, using each city’s daily mortality, pollutant concentrations, and meteorological data aggregated at the city level. All statistical analyses were performed using R version 4.2.3; Supplementary Figure S1 was generated using ArcGIS version 10.8. The overall analytical workflow is summarized in Figure 1.

#### 2.2.3 Step 2-model validation and performance comparison

To evaluate the performance and generalizability of the constructed indices, we conducted a series of validation analyses that were entirely independent of the index construction phase. These included assessments of model fit and predictive performance, comparison of risks between the two indices, and evaluation of extreme temperature risk adjustment. For these validation comparisons, the indices were categorized into quartiles and modeled using Poisson regression to quantify excess risks across discrete risk categories. The same validation protocol was applied independently to Ningbo and London.

For the quartile-based risk comparison, we used the first group (lowest quartile) as the reference to estimate odds ratios and excess risks for each higher risk category (40, 41). Additionally, to visualize the adjustment of the TS-AQHI, the standard AQHI to the effect of daily risk warnings, we created a time series plot. The TS-AQHI and the standard AQHI use the same hazard classification, and in order to visualize the risk of death more closely, we have used a standardized methodology that rescaled the number of deaths per day to a range of 0–10, which is in line with the range of the AQHI.

To better explore the difference between the new and old indices, especially in terms of risk adjustment for extreme temperatures, we performed *t*-tests on maximum and average daily temperatures for the same risk groups of the old and new indices according to the standardized grading.

#### 2.2.4 Step 3-exploratory subgroup analyses

To explore whether the association between environmental exposures and mortality varied across population subgroups, we conducted age- and sex-stratified analyses using the same GAM framework. Daily mortality counts were stratified into two age groups (<65 years and ≥65 years) and two gender groups (male and female). These analyses were restricted to Ningbo, where stratified mortality data were available.

## 3 Results

### 3.1 Trends of air pollution, heat, and mortality

Descriptive statistics of daily deaths, air pollutants, and weather conditions are summarized in Tables 1, 2. Annual trends of air pollutants and weekly mean temperature are shown in Supplementary Figure S2. Specifically, statistical analysis of Ningbo from 2014 to 2017 indicates that the concentrations of major air pollutants, including PM_10_, PM_2.5_, SO_2_, NO_2_, and CO showed a significant decreasing trend year by year. In contrast, ground-level ozone (O₃) concentrations exhibited a marked upward trend, with its annual average surpassing the national Grade I standard in 2017 (42). However, despite the decline in pollutant levels, the overall mortality rate did not decrease as anticipated and instead remained relatively stable during the study period. This pattern contrasts with the decreasing trends of most air pollutants but corresponds closely with variations in the temperature. Meanwhile, statistical data from London reveal a similar relationship between mortality trends and pollutant variations as observed in Ningbo. From 2014 to 2017, temperature variability intensified in both Ningbo and London. Ningbo’s peak weekly summer mean temperature rose by approximately 4 °C to 32 °C, whereas London’s extreme winter minimum dropped by nearly 2 °C to 3.4 °C, reflecting a growing non-stationary climate regime marked by increasing temperature extremes.

**Table 1:** Descriptive statistics on daily counts of deaths, air pollution and weather conditions in Ningbo, 2014–2017.

Variables					
Description	2014	2015	2016	2017	*p*-value
Number of daily deaths Median (Q1, Q3)					
Total	97 (88, 112)	103 (92, 117)	102 (90, 119)	99 (86, 111)	<0.001
Gender					
Male	56 (48, 63)	58 (51, 66)	58 (51, 66)	55 (48, 63)	<0.001
Female	42 (37, 50)	45 (39, 52)	44.5 (38, 52)	42 (35, 50)	<0.001
Age					
≥65	73 (64, 86)	78 (69, 93)	78 (69, 93)	77 (67, 89)	<0.001
18–65	23 (19, 26)	23 (19, 26)	22 (18, 25)	19 (15, 23)	<0.001
<18	1 (0, 2)	1 (0, 2)	0 (0, 2)	0 (0, 2)	<0.05
Air pollution Mean+_SD					
PM_10_ (μg/m^3^)	70.94 ± 38.20	69.41 ± 40.78	62.01 ± 33.03	59.75 ± 32.53	<0.001
PM_2.5_ (μg/m^3^)	45.26 ± 24.85	44.67 ± 29.22	38.56 ± 22.90	36.88 ± 22.60	<0.001
SO_2_ (μg/m^3^)	17.03 ± 8.88	15.51 ± 7.21	12.75 ± 4.57	10.16 ± 3.59	<0.001
NO_2_ (μg/m^3^)	40.52 ± 16.91	42.79 ± 17.88	39.17 ± 16.83	38.32 ± 17.67	<0.001
CO (mg/m^3^)	0.94 ± 0.26	0.89 ± 0.26	0.80 ± 0.21	0.78 ± 0.19	<0.001
O_3_ (μg/m^3^)	91.97 ± 37.34	96.31 ± 42.35	94.33 ± 40.04	100.21 ± 42.62	<0.05
Weather conditions Mean ± SD					
Mean daily temperature (°C)	17.21 ± 8.17	17.17 ± 7.98	17.94 ± 8.63	17.78 ± 8.77	0.64
RH (%)	79.06 ± 10.74	79.91 ± 11.15	80.93 ± 11.13	78.79 ± 12.27	0.013

**Table 2:** Descriptive statistics on daily counts of deaths, air pollution and weather conditions in London, 2014–2017.

Variables					
Description	2014	2015	2016	2017	*p*-value
Number of daily total deaths Median (Q1, Q3)	132 (126, 143)	133 (122, 144)	133 (124, 144)	132 (124, 148)	<0.001
Air pollution Mean+_SD					
PM_10_ (μg/m^3^)	23.79 ± 10.28	19.78 ± 10.80	17.09 ± 11.21	14.15 ± 7.94	<0.001
PM_2.5_ (μg/m^3^)	15.53 ± 7.89	12.19 ± 9.76	12.04 ± 10.31	9.16 ± 6.80	<0.001
SO_2_ (μg/m^3^)	8.82 ± 3.11	2.37 ± 1.43	2.34 ± 1.10	1.45 ± 0.62	<0.001
NO_2_ (μg/m^3^)	87.51 ± 26.29	34.92 ± 18.34	32.70 ± 16.53	29.20 ± 12.55	<0.001
CO (mg/m^3^)	0.50 ± 0.15	0.23 ± 0.15	0.16 ± 0.11	0.15 ± 0.05	<0.001
O_3_ (μg/m^3^)	15.81 ± 9.97	37.23 ± 18.31	39.75 ± 18.52	44.44 ± 19.73	<0.001
Weather conditions Mean ± SD					
Mean daily temperature (°C)	12.36 ± 4.61	12.11 ± 5.45	12.26 ± 5.38	12.52 ± 6.08	0.88
RH (%)	72.31 ± 9.67	75.02 ± 9.78	75.83 ± 9.76	74.01 ± 11.10	<0.001

### 3.2 Two-City independent validation results

City-specific pollutant weights and the distributional characteristics of AQHI and TS-AQHI are provided in Supplementary Tables S3, S4, respectively, with TS-AQHI demonstrating systematically higher values and broader exposure ranges compared to the conventional index.

#### 3.2.1 Dose–response relationship across risk levels

**Figure 2:**
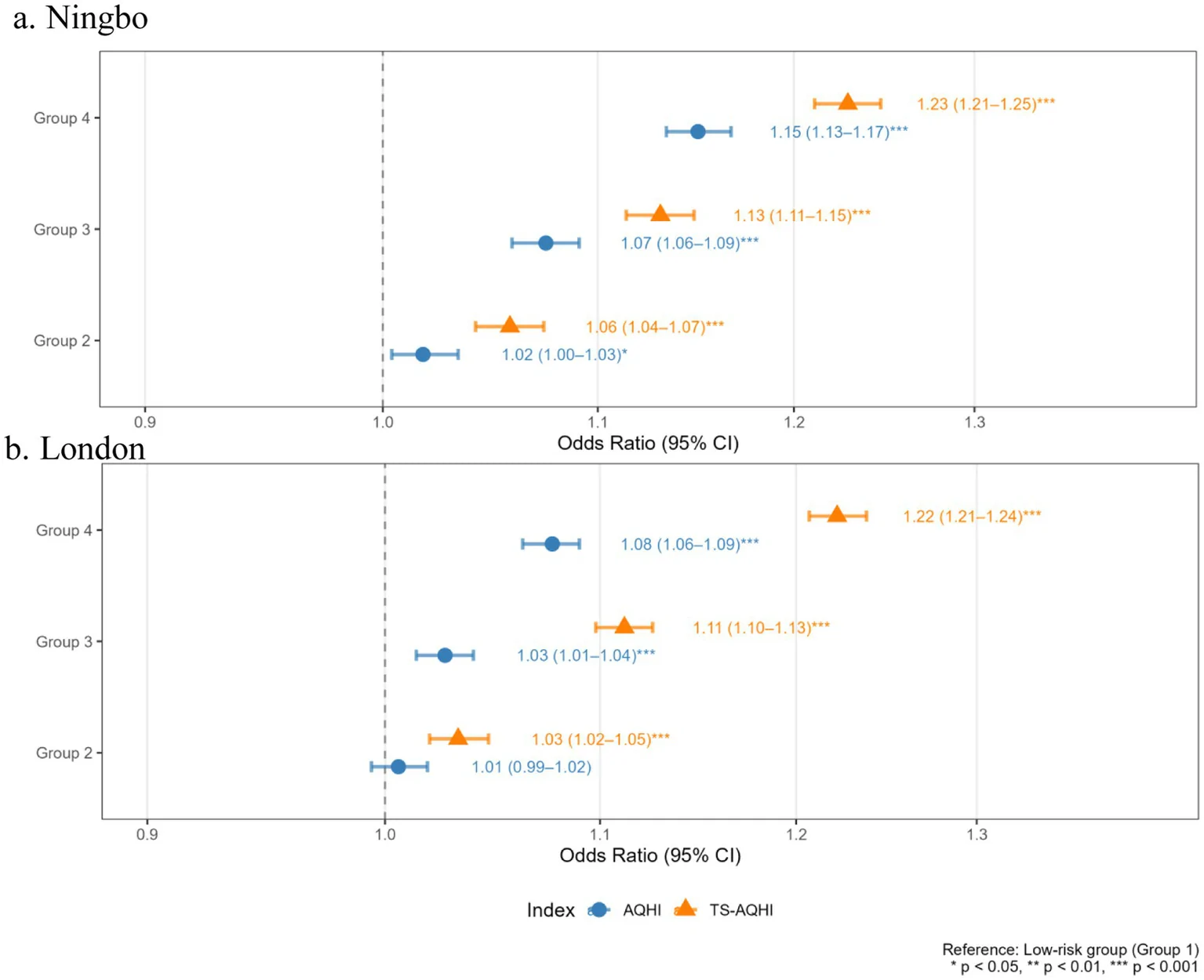
AQHI and TS-AQHI related hazard ratios per elevated risk group, compared with low-risk groups in London and Ningbo: **(a)** Ningbo; **(b)** London. Group2, Group3, and Group4 correspond to index values in the second (25th–50th percentile), third (50th–75th percentile), and fourth (75th–100th percentile) quartiles, representing low-to-moderate, moderate-to-high, and highest exposure categories, respectively. The first quartile (0–25th percentile) is the reference group.

Table 3 and Figure 2a present the regression analysis of categorized risk indices in Ningbo, using the low-risk group as reference, demonstrating a consistent and statistically significant dose–response relationship with mortality outcomes (all trend tests *p* < 0.001). Both indices showed monotonically increasing odds ratio (OR) and ER across ascending risk categories, confirming the validity of the index grouping approach. The comparative analysis demonstrated superior risk discrimination by TS-AQHI relative to conventional AQHI. In the comparison between the serious group and the low group, the TS-AQHI model yielded an OR of 1.23 (95% CI: 1.21, 1.25), exceeding the conventional AQHI’s OR of 1.15 (95% CI: 1.13, 1.17). Similarly, the excess risk estimate for TS-AQHI was 22.9 (95% CI: 21.1, 24.7), substantially higher than the 15.0 (95% CI: 13.4, 16.7) observed for conventional AQHI. These differences represent a 7.0% relative improvement in OR and 52.7% increase in ER magnitude (all *p*-values <0.001), indicating that the TS-AQHI predicted more accurately in the high-risk group. Notably, while the conventional AQHI reached statistical significance only at the borderline for group 2 (*p* = 0.015), the TS-AQHI demonstrated robust significance even at lower risk levels (*p* < 0.001).

**Table 3:** Excess mortality risk and hazard ratios by AQHI and TS-AQHI risk groups, compared with low-risk groups, Ningbo, 2014–2017.

Variable	OR (95% CI)	ER (95% CI)	*p*-value
AQHI			
(Intercept)	97.39 (96.37, 98.42)	9,639.1 (9,537.4, 9741.9)	<0.001
AQHI.group2	1.02 (1.00, 1.03)	1.8 (0.3, 3.4)	0.015
AQHI.group3	1.07 (1.06, 1.09)	7.5 (5.9, 9.1)	<0.001
AQHI.group4	1.15 (1.13, 1.17)	15.0 (13.4, 16.7)	<0.001
TS-AQHI			
(Intercept)	93.50 (92.50, 94.50)	9,249.6 (9,149.8, 9350.4)	<0.001
TS-AQHI.group2	1.06 (1.04, 1.07)	5.8 (4.2, 7.4)	<0.001
TS-AQHI.group3	1.13 (1.11, 1.15)	13.1 (11.4, 14.8)	<0.001
TS-AQHI.group4	1.23 (1.21, 1.25)	22.9 (21.1, 24.7)	<0.001

The intercept represents the baseline outcome level when all predictors are at reference levels. AQHI.group2, AQHI.group3, and AQHI.group4 correspond to AQHI values in the second (25th–50th percentile), third (50th–75th percentile), and fourth (75th–100th percentile) quartiles, representing low-to-moderate, moderate-to-high, and highest exposure categories, respectively. The first quartile (0–25th percentile) is the reference group.

Clinically, these metrics translate to important risk differences: the TS-AQHI’s OR of 1.23 indicates a 23.0% elevated mortality risk in the highest-risk group compared to the reference, while the ER of 22.9 corresponds that each one-unit increase in TS-AQHI is associated with a 22.9% increase in daily mortality. The consistently narrower confidence intervals for TS-AQHI parameters further suggest enhanced estimation precision. This systematic upward shift across the entire distribution indicates that TS-AQHI provides a more sensitive assessment of health risks, potentially capturing additional mortality risks that conventional AQHI metrics may underestimate. This finding is also consistent with the results observed in London. Specifically, the Poisson regression model assessing risk level changes in the index (Table 4) demonstrated that the high-risk group had a significantly higher excess risk of death than the low-risk group (used as the reference) across all metrics. Notably, the conventional AQHI also lost statistical significance in the second-risk group in London (*p* = 0.335), mirroring the instability observed in the current Ningbo cohort. This consistency supports the adequacy of the index as a risk predictor. Furthermore, the ER for the high-risk group for the TS-AQHI is 22.2 (95% CI: 20.7, 23.8) higher than 7.7 (95% CI: 6.3, 9.0) for the AQHI, Figure 2b.

**Table 4:** Excess mortality risk and hazard ratios by AQHI and TS-AQHI risk groups, compared with low-risk groups, London, 2014–2017.

Variable	OR (95% CI)	ER (95% CI)	*p*-value
AQHI			
(Intercept)	130.61 (129.44, 131.80)	12,961.3 (12,844.1, 13079.6)	<0.001
AQHI.group2	1.01 (0.99, 1.02)	0.6 (−0.6, 1.9)	0.335
AQHI.group3	1.03 (1.01, 1.04)	2.7 (1.4, 4.0)	<0.001
AQHI.group4	1.08 (1.06, 1.09)	7.7 (6.3, 9.0)	<0.001
TS-AQHI			
(Intercept)	122.89 (121.76, 124.04)	12,189.5 (12,075.8, 12,304.2)	<0.001
TS-AQHI.group2	1.03 (1.02, 1.05)	3.3 (2.0, 4.7)	<0.001
TS-AQHI.group3	1.11 (1.10, 1.13)	11.2 (9.8, 12.6)	<0.001
TS-AQHI.group4	1.22 (1.21, 1.24)	22.2 (20.7, 23.8)	<0.001

The intercept represents the baseline outcome level when all predictors are at reference levels. AQHI.group2, AQHI.group3, and AQHI.group4 correspond to AQHI values in the second (25th–50th percentile), third (50th–75th percentile), and fourth (75th–100th percentile) quartiles, representing low-to-moderate, moderate-to-high, and highest exposure categories, respectively. The first quartile (0–25th percentile) is the reference group.

#### 3.2.2 Time-series alignment with mortality

**Figure 3:**
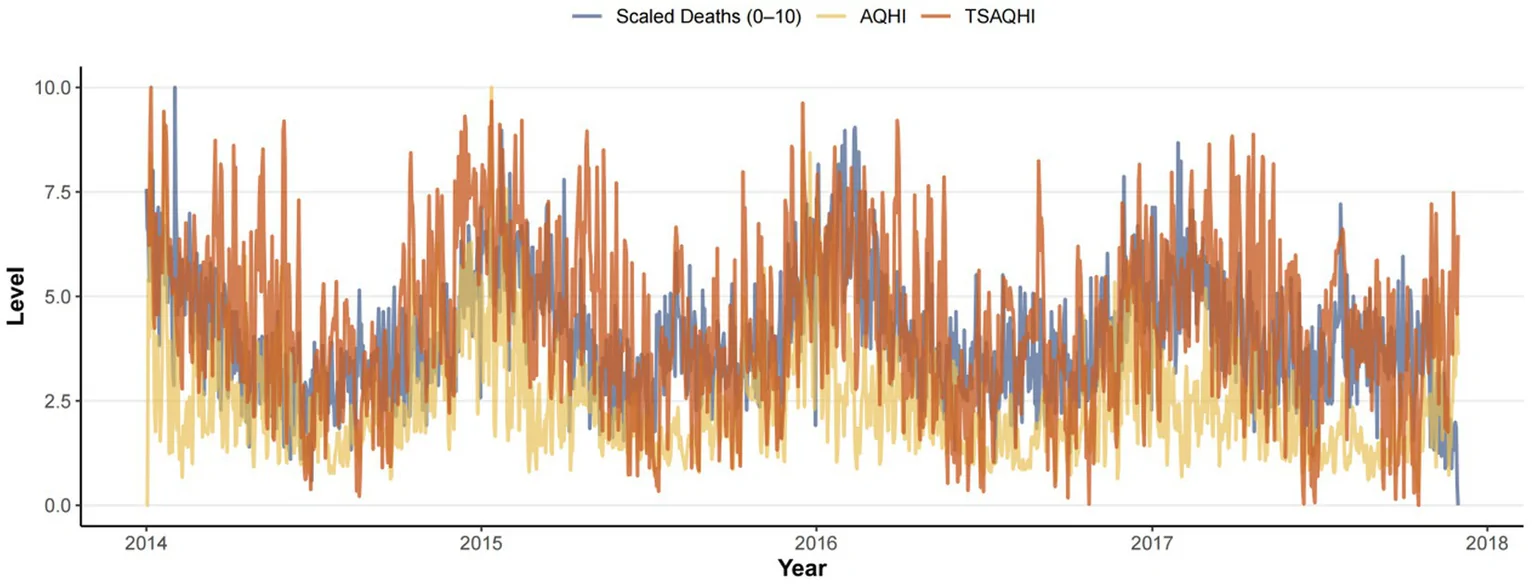
Time series comparison of AQHI, TS-AQHI distance with deaths grading in Ningbo, 2014–2017.

**Figure 4:**
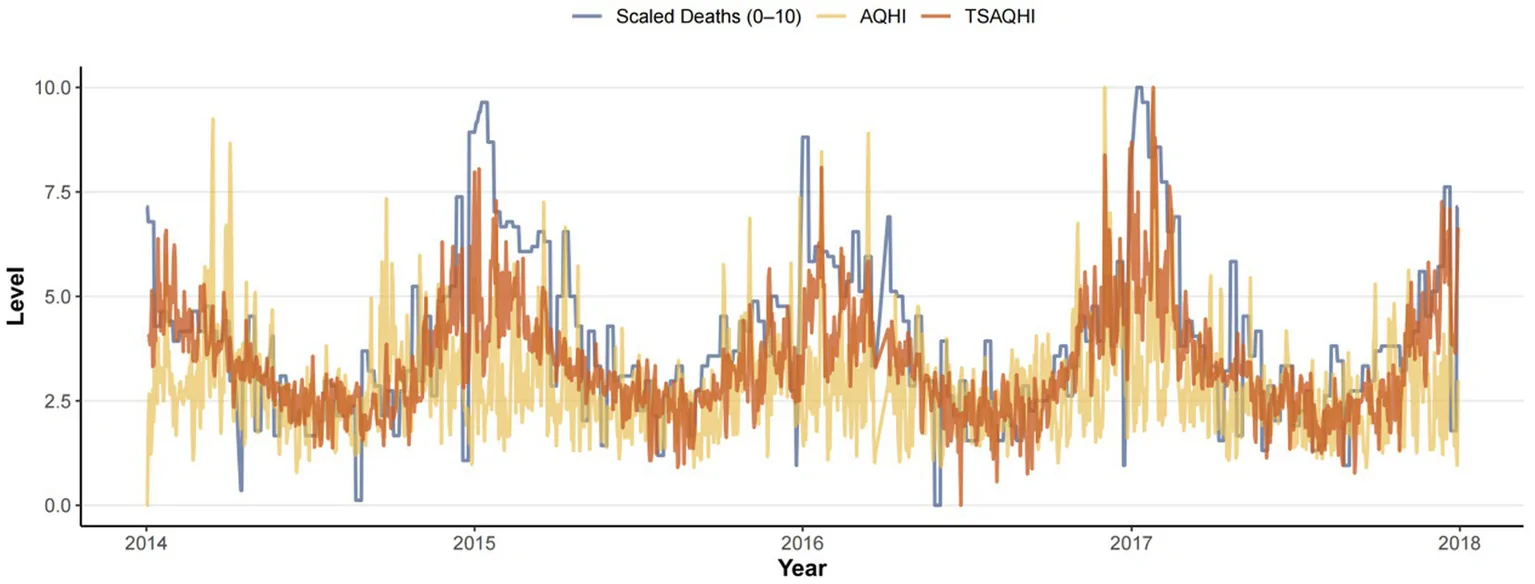
Time series comparison of AQHI, TS-AQHI distance with deaths grading in London, 2014–2017.

Figure 3 and Figure 4 show the adjustment effect of TS-AQHI on the AQHI index. The comparative analysis included the number of deaths, scaled to 0–10. The composite index TS-AQHI was also graded using the same criteria as the AQHI index. To facilitate visual analysis, the time series was plotted. As the prediction becomes more accurate, i.e., the smaller the difference between deaths and the AQHI or TS-AQHI, the closer the line becomes. First, all three metrics (AQHI, TS-AQHI, and scaled mortality) exhibit pronounced seasonal variability, consistently showing peak mortality risks during winter months, secondary summer peaks, and lowest risk periods in spring and autumn. This seasonal synchrony confirms the indices’ basic validity in capturing annual risk patterns. Second, the TS-AQHI demonstrates superior predictive accuracy, evidenced by closer alignment between its trendline and actual mortality data. Particularly during winter high-risk periods (December–January), TS-AQHI shows enhanced sensitivity, better capturing the magnitude and temporal patterns of mortality spikes compared to AQHI. Third, TS-AQHI demonstrates improved calibration during summer months, effectively adjusting for the characteristic secondary risk peak while maintaining accurate baseline risk assessment during transitional seasons.

The consistent performance across both cities suggests that TS-AQHI’s incorporation of temperature provides robust enhancements over conventional AQHI in different climatic and pollution contexts. Crucially, this cross-regional validation reveals TS-AQHI’s enhanced capacity to detect winter mortality spikes through superior sensitivity to cold-weather risks while simultaneously providing more precise calibration of summer heat-related risk elevations. These replicated findings in climatically distinct urban environments confirm TS-AQHI’s generalizability as an advanced health risk forecasting tool, with its predictive advantages persisting across diverse meteorological and pollution regimes. The replication of findings in two distinct urban environments strengthens the evidence for adopting TS-AQHI as a more refined approach for public health risk communication and intervention planning.

#### 3.2.3 Comparative risk classification

**Figure 5:**
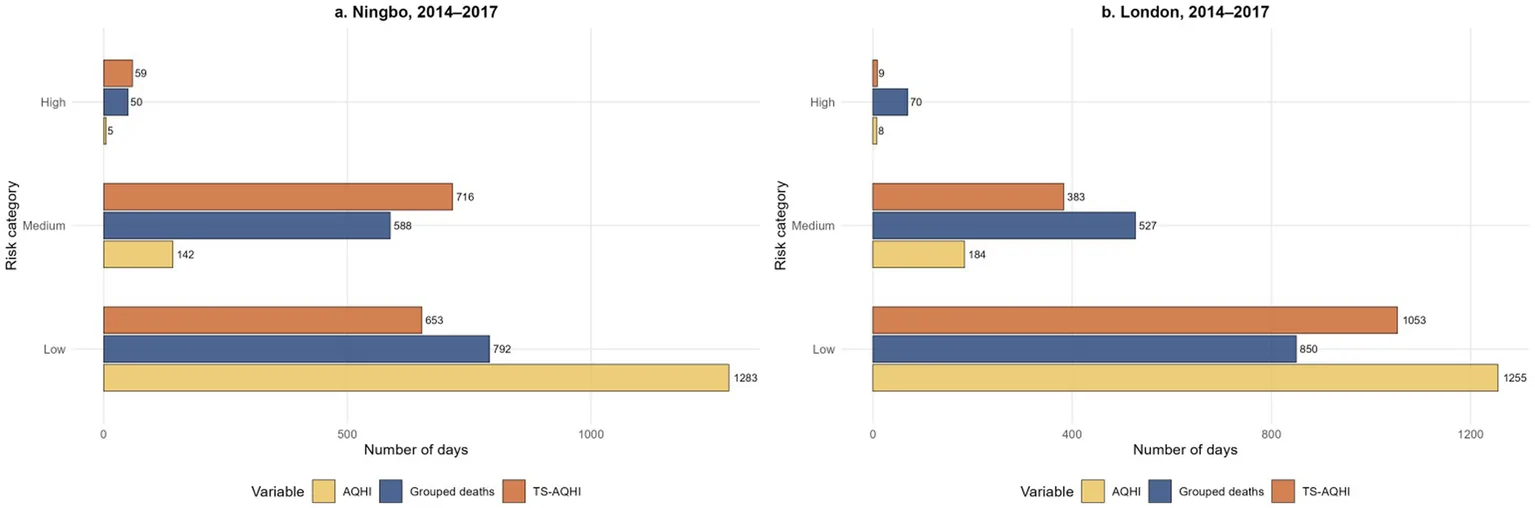
Health risk categorization for two indices for the period 2014–2017 in London and Ningbo: **(a)** Ningbo; **(b)** London. Daily all cause mortality was grouped into three ordinal categories: Low (< mean), Medium (mean to < mean +2SD), and High (≥mean+2SD). The horizontal axis indicates the number of days and the vertical axis is risk ategorization.

Figure 5 compares the health risk classification results of the two indices in London and Ningbo from 2014 to 2017. The traditionally established AQHI exhibited a pronounced right-skewed distribution in Ningbo, classifying 89.8% (1,283 days) as low-risk, 9.9% (142 days) as moderate-risk, and only 0.35% (5 days) as high-risk. In contrast, TS-AQHI demonstrated substantially enhanced risk discrimination, reducing low-risk days to 45.7% while increasing moderate-risk days to 50% and high-risk days to 59 days (4%). Similar improvements were observed in London, where TS-AQHI decreased low-risk days from 86.7 to 72.8% (1,053 days) and increased moderate-risk days from 12.7% (184 days) to 26.5% (383 days). These classification differences were statistically confirmed by Wilcoxon signed-rank tests (*p* < 0.05). TS-AQHI consistently classifies a higher proportion of days into the moderate- and high-risk categories compared to the conventional AQHI, and its distribution more closely follows the mortality-based reference, particularly for the high-risk category.

A *t*-test was performed on the AQHI and TS-AQHI to compare the mean daily maximum temperature and daily average temperature of different risk level groups (Table 5). It is worth noting that the *t*-test results showed that in Ningbo, TS-AQHI applied significantly warmer criteria for both moderate- and low-risk groups. For the moderate-risk group, TS-AQHI raised temperature thresholds (max: 15.12 °C to 20.24 °C, mean: 10.16 °C to 15.54 °C; both *p* < 0.001), while showing a similar warming trend for low-risk groups (22.95 °C vs. 24.56 °C for max, 18.58 °C vs. 20.63 °C for mean; both *p* < 0.001). London exhibited a more nuanced pattern: TS-AQHI significantly increased the threshold for low-risk groups (max: 16.18 °C to 18.06 °C, mean: 12.66 °C to 14.32 °C; both *p* < 0.001), but contrary to Ningbo, it significantly lowered the thresholds for moderate-risk groups (max: 13.37 °C to 9.80 °C, mean: 10.42 °C to 7.09 °C; both *p* < 0.001).

**Table 5:** AQHI and TS-AQHI *t*-tests of median daily maximum temperature and daily average temperature for different risk class groups, Ningbo and London, 2014–2017.

Risk group	AQHI	TS-AQHI	*p*-value
Ningbo			
High risk			
Maximum daily temperature (°C)	9.58	18.09	<0.001
Mean daily temperature (°C)	5.50	11.98	<0.001
Mid risk			
Maximum daily temperature (°C)	15.12	20.24	<0.001
Mean daily temperature (°C)	10.16	15.54	<0.001
Low risk			
Maximum daily temperature (°C)	22.95	24.56	<0.001
Mean daily temperature (°C)	18.58	20.63	<0.001
London			
High risk			
Maximum daily temperature (°C)	11.99	8.04	0.09
Mean daily temperature (°C)	8.07	5.27	0.13
Mid risk			
Maximum daily temperature (°C)	13.37	9.80	<0.001
Mean daily temperature (°C)	10.42	7.09	<0.001
Low risk			
Maximum daily temperature (°C)	16.18	18.06	<0.001
Mean daily temperature (°C)	12.66	14.32	<0.001

These directional adjustments demonstrate TS-AQHI’s enhanced capacity to more accurately identify hazardous thermal exposures through its interaction with the temperature, exhibiting both regionally adaptive threshold modifications rather than uniform stringency and improved differentiation of marginal risk days in low-risk categories, while effectively adapting to regional climate variations, particularly evident in its systematic modulation of temperature thresholds across both cities.

#### 3.2.4 Seasonal analysis

**Figure 6:**
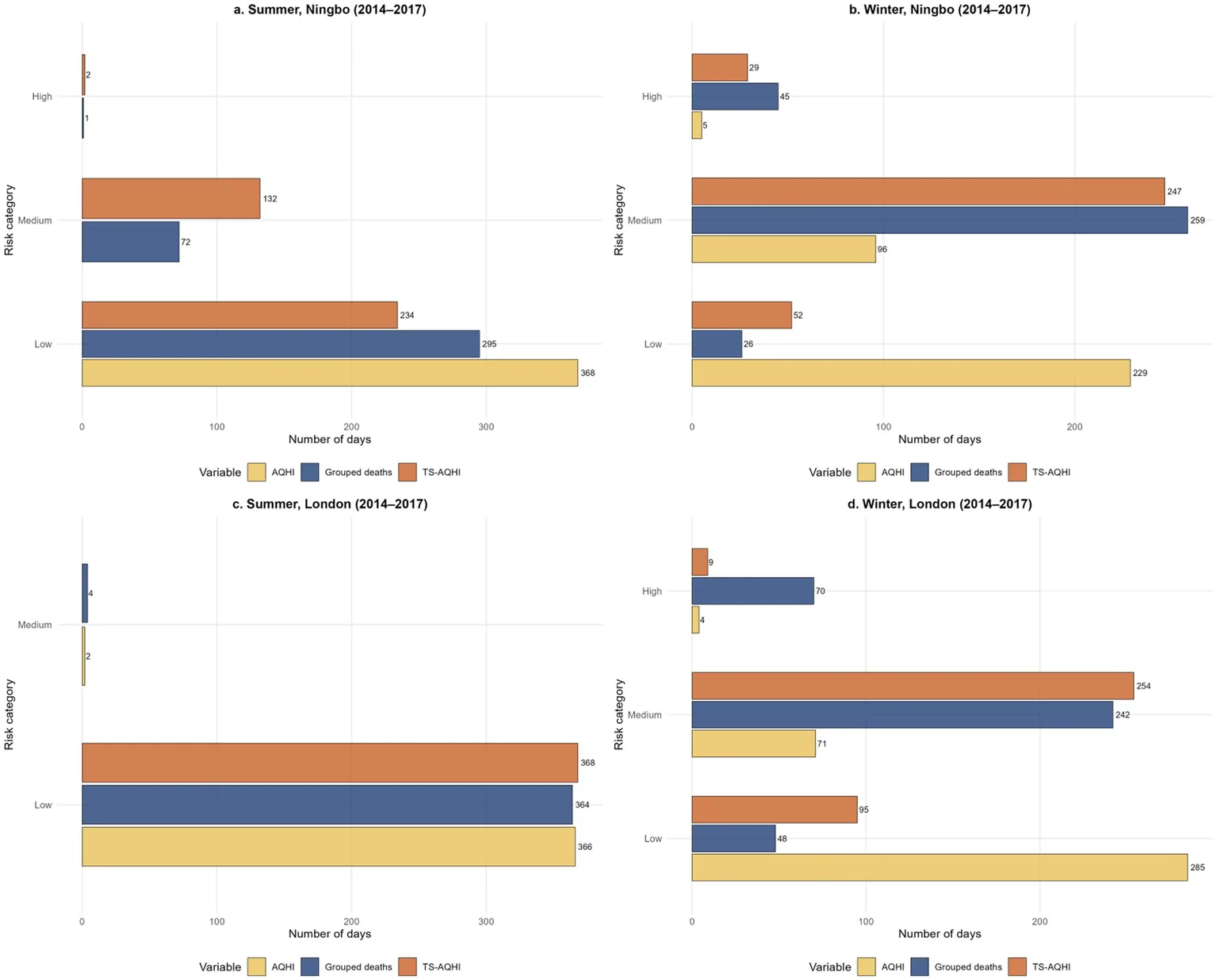
Health risk categorization for two indices for the period 2014–2017 in Ningbo, summer and winter: **(a)** Summer, Ningbo; **(b)** Winter, Ningbo; **(c)** Summer, London; **(d)** Winter, London. Daily all-cause mortality was grouped into three ordinal categories: Low (< mean), Medium (mean to <mean + 2 SD), and High (≥ mean + 2 SD). The horizontal axis indicates the number of days and the vertical axis is risk categorization.

Seasonal analysis revealed critical temperature-dependent risk assessment patterns. As shown in Figure 6, consistent with the mortality-based reference, TS-AQHI issued substantially more moderate-to-high-risk warnings during both summer heatwaves and winter cold spells than the conventional AQHI. In Ningbo, TS-AQHI identified 132 moderate-risk days during summer, whereas the conventional AQHI failed to flag any days above the low-risk category. In winter, TS-AQHI issued warnings for 276 days (29 high + 247 medium), representing a 2.7-fold increase compared to the 101 days (5 high + 96 medium) identified by AQHI. Similarly, in London, TS-AQHI increased winter warnings from 75 to 263 days (9 high + 254 medium). In both summer and winter, the TS-AQHI performed better than the AQHI in the moderate-to-high-risk warning zones.

During London winters (December–February), TS-AQHI’s low-risk group showed higher mean daily temperatures (8.85 °C vs. 6.94 °C, *p* < 0.001), indicating better recognition of temperature-moderated health risks, while its moderate-risk group captured more cold-exposure days through lower maximum temperatures (8.86 °C vs. 9.64 °C, *p* < 0.1) and mean temperatures (7.29 °C vs. 6.34 °C, *p* < 0.05) (Table 6). Differences were also observed in Ningbo (Table 7). In winter, the TS-AQHI high-risk group exhibited significantly higher maximum (12.49 °C vs. 9.58 °C, *p* < 0.1) daily temperatures compared with AQHI, whereas the TS-AQHI medium-risk group showed lower maximum (11.75 °C vs. 12.66 °C, *p* < 0.1) temperatures. In summer, TS-AQHI low-risk group showed lower maximum (30.33 °C vs. 31.30 °C, *p* < 0.05) temperatures and mean (26.26 °C vs. 27.02 °C, *p* < 0.05) temperatures. This indicates that TS-AQHI more sensitively identified winter low-temperature health risks by reclassifying a greater proportion of cold days into the moderate-risk category (Table 7). The reconstructed temperature-response curves revealed more epidemiologically plausible risk gradients, particularly in winter.

**Table 6:** AQHI and TS-AQHI *t*-tests of median daily maximum temperature and daily average temperature for different risk class groups in London, summer and winter, 2014–2017.

Risk group	AQHI	TS-AQHI	*p*-value
Winter			
High risk			
Maximum daily temperature (°C)	7.88	8.04	0.924
Mean daily temperature (°C)	4.84	5.27	0.8
Mid risk			
Maximum daily temperature (°C)	9.64	8.86	0.0672
Mean daily temperature (°C)	7.29	6.34	0.0293
Low risk			
Maximum daily temperature (°C)	9.45	11.21	<0.001
Mean daily temperature (°C)	6.94	8.85	<0.001
Summer			
Low risk			
Maximum daily temperature (°C)	22.31	22.30	0.969
Mean daily temperature (°C)	18.09	18.09	0.994

The sample sizes in the summer high-risk and mid-risk groups were too small to perform reliable *t*-tests.

**Table 7:** AQHI and TS-AQHI *t*-tests of median daily maximum temperature and daily average temperature for different risk class groups in Ningbo, summer and winter, 2014–2017.

Risk group	AQHI	TS-AQHI	*p*-value
Winter			
High risk			
Maximum daily temperature (°C)	9.58	12.49	0.0537
Mean daily temperature (°C)	5.50	5.51	0.996
Mid risk			
Maximum daily temperature (°C)	12.66	11.75	0.0929
Mean daily temperature (°C)	7.33	7.05	0.509
Low risk			
Maximum daily temperature (°C)	11.27	10.42	0.2
Mean daily temperature (°C)	6.79	7.21	0.46
Summer			
Low risk			
Maximum daily temperature (°C)	31.30	30.33	<0.05
Mean daily temperature (°C)	27.02	26.26	<0.05

The sample sizes in the summer high-risk and mid-risk groups were too small to perform reliable *t*-tests.

### 3.3 Exploratory age- and sex-stratified analyses in Ningbo

In exploratory subgroup analyses restricted to Ningbo, TS-AQHI yielded higher explained deviance than AQHI across all strata. Among individuals aged ≥ 65 years, TS-AQHI explained 25.1% of mortality variance compared with 20.5% for AQHI alone. In those aged < 65 years, both indices showed significant but weak associations, with comparable explanatory power (*R^2^* = 8.2%). Gender-stratified analysis further confirmed the added value of TS-AQHI. For females, TS-AQHI explained 20.3% of mortality variance versus 16.0% for AQHI. For males, TS-AQHI also improved model performance (*R^2^* = 18.9% vs. 15.6%) (Supplementary Table S5).

Together, these descriptive findings suggest that incorporating temperature into AQHI may yield greater gains in explanatory power among older adults and females, though the absence of formal interaction testing limits causal interpretation. Results should be considered hypothesis-generating pending confirmation in multi-city studies with adequate statistical power.

## 4 Discussion

### 4.1 Core innovation: capturing synergistic effects through interaction modeling to correct systematic risk classification bias

The conventional AQHI is built upon an additive weighting assumption of multiple pollutant effects, derived from earlier time-series mortality studies that modeled pollutants independently or as linear sums (15, 17). TS-AQHI departs from this paradigm by explicitly modeling the synergy between temperature and air pollution through a multiplicative interaction term. This allows the index to capture amplified mortality risks on days when both stressors co-occur. Under additive models, these excess deaths are systematically underestimated. This superiority in risk classification is grounded in both mechanistic and statistical reasoning. Mechanistically, heat stress and pollutant exposure share converging biological pathways. Both induce systemic oxidative stress, activate pulmonary and systemic inflammatory cascades, and impair autonomic cardiovascular regulation. When exposures coincide, these processes become mutually reinforcing (13, 43). Statistically, the multiplicative interaction term in the GAM framework permits the effect of pollution on mortality to vary as a function of temperature, and vice versa. This relaxes the restrictive additivity assumption imposed by conventional AQHI. Large-scale multi-city studies have provided empirical support for this approach. An analysis of 620 cities across 36 countries found that high temperatures significantly enhance the mortality effects of PM_10_ and O_3_, with interaction effects consistently exceeding additive predictions (11). Similar synergistic patterns have been reported for cold temperatures and particulate matter in multi-city settings (12). A systematic review of 62 studies further confirmed interactive effects between high temperatures and O_3_, PM_10_, and PM_2.5_ on mortality, with the strongest evidence for cardiovascular and respiratory outcomes (13). By embedding such interaction directly into the index structure, TS-AQHI’s risk stratification aligns more closely with actual mortality patterns. This is particularly true on days when extreme temperatures coincide with elevated pollution. These are precisely the conditions under which conventional AQHI’s additive framework most severely underestimates health risks.

### 4.2 Context-specific performance across climates: threshold self-adaptation mechanisms of TS-AQHI

The directionally divergent temperature threshold adjustments observed between Ningbo and London substantiate TS-AQHI’s capacity for data-driven self-adaptation to locally dominant climatic risk drivers. This adaptation does not represent a uniform tightening of warning criteria across all settings. In Ningbo, which has a subtropical climate, TS-AQHI significantly raised temperature thresholds for moderate- and low-risk groups. This indicates that the model identified summer heat as the predominant additional driver of health risk in this region. In London, which has a temperate maritime climate, TS-AQHI significantly lowered thresholds for the moderate-risk group. This opposite direction reflects the greater health burden imposed by winter cold exposure in this city.

This directional, empirically derived differentiation is consistent with the well-established U-shaped or J-shaped relationship between temperature and mortality. The location and steepness of this risk curve vary substantially across climatic zones (7, 8). A multi-country study across 13 countries found that cold-related mortality far exceeded heat-related mortality globally, but with wide regional variation (6). Heat effects were more pronounced in cooler climates, while cold effects dominated in warmer regions. These patterns reflect differential acclimatization and behavioral adaptation across populations (44). A multi-city analysis of 66 Chinese communities further confirmed that south China had higher minimum mortality temperatures than north China, with larger cold effects in the south and more pronounced hot effects in the north (45). These regional disparities underscore the need for climate-adaptive warning systems that account for local temperature-mortality profiles.

TS-AQHI’s adaptive calibration is particularly valuable in the context of climate change. The frequency and intensity of both heatwaves and cold spells are increasing asymmetrically across regions (9). Rather than relying on externally imposed, uniform temperature cutoffs, TS-AQHI derives its thresholds empirically from local data. This allows the model to automatically identify region-specific heat-sensitive and cold-sensitive periods based on each city’s characteristic temperature-mortality response profile. This flexibility offers public health agencies a context-sensitive tool to tailor early warnings to local climatic conditions. Such a capability addresses a critical need identified in recent reviews, which have called for climate-adaptive public health warning systems (13, 36).

### 4.3 Effect modification and model boundaries: interpretability of subgroup patterns and inherent limitations

The age- and sex-stratified analyses conducted in Ningbo suggested that TS-AQHI yielded greater improvements in model explanatory power among older adults (≥65 years) compared with the conventional AQHI. This finding aligns with established epidemiological evidence that older adult populations exhibit heightened vulnerability to both air pollution and temperature extremes (46, 47). Physiologically, age-related declines in thermoregulatory capacity compound the effects of pollutant-induced oxidative stress and inflammation. These declines include reduced sweating efficiency, impaired vasodilation, and diminished cardiovascular reserve. The synergistic impact of combined exposures is therefore particularly deleterious in this subgroup (36, 48). The gender patterns observed in our study, though more modest, are consistent with emerging literature documenting sex-specific differences in environmental health responses. These differences may reflect multiple underlying factors. Hormonal influences on cardiovascular reactivity, differences in lung function and airway morphology, and sex-specific patterns of time-activity and occupational exposure all potentially contribute to the observed disparities (6, 49).

More importantly, across all subgroups, environmental exposure variables explained no more than 25% of daily mortality variance. This holds true even with the inclusion of temperature interaction terms. This observation delineates a crucial scientific boundary. While air pollution and temperature are important environmental risk factors at the population level, they account for only a modest fraction of mortality fluctuations. The majority of variance is determined by individual-level factors including chronic disease status, frailty, socioeconomic position, healthcare accessibility, and genetic susceptibility (4, 14). This does not diminish the value of TS-AQHI, but rather clarifies its appropriate scope. As with other environmental health indices, TS-AQHI is most suitably applied as a population-level early warning tool for guiding public health communications and interventions. It should not be viewed as a substitute for individualized clinical risk assessment (18, 22). The subgroup findings, while hypothesis-generating, warrant cautious interpretation and require confirmation in future studies designed to test interaction effects.

### 4.4 Limitations and future directions

Several limitations should be acknowledged. First, our reliance on city-level aggregated data may not capture individual-level exposure differences, though this aligns with the practical need for a warning index that uses publicly available monitoring data for timely population-level alerts. The consistent findings across Ningbo and London support the generalizability of this approach. Second, the WQS regression assumes a weighted sum of pollutant effects, which may not fully capture complex non-linear interactions; future work could explore Bayesian kernel machine regression or similar mixture modeling techniques. Third, while TS-AQHI incorporates a temperature-pollution interaction term, it still models the temperature-mortality relationship linearly and therefore cannot fully characterize the documented U-shaped or threshold-type risk curves, especially at temperature extremes (7, 8, 11). This simplification was chosen to maintain computational feasibility and cross-city transferability, but future research could embed distributed lag nonlinear models or non-parametric smoothing to better capture nonlinear effects across climates (12, 13, 38). Finally, validation in additional cities with varying pollution profiles, demographics, and healthcare systems is needed to further establish the index’s broader applicability.

## 5 Conclusion

This study develops and validates a transferable Temperature-Sensitive Air Quality Health Index that explicitly accounts for the synergistic interaction between thermal stress and multi-pollutant exposure-a dimension systematically overlooked by conventional additive indices. Methodologically, the core innovation lies in embedding a multiplicative interaction term within a GAM-based framework, which enables the index to dynamically capture amplified mortality risks when extreme temperatures and elevated pollution co-occur, rather than assuming independent effects. From a public health practice perspective, TS-AQHI offers a more sensitive and context-appropriate tool for early warning systems. It substantially improves risk stratification during both summer heatwaves and winter cold spells, correcting the systematic underestimation inherent in existing indices and providing public health agencies with more timely signals for triggering interventions and health communications. This adaptive calibration to local climatic conditions enhances the index’s utility across diverse geographical settings. However, as an index derived from city-level aggregated data, TS-AQHI is designed for population-level early warning and should not be misapplied as a tool for individualized risk prediction; further validation across a broader range of cities is needed to confirm its generalizability.

## Data Availability

Publicly available datasets were analyzed in this study. This data can be found here: National Science and Technology Basic Resources Survey Program (Project: “Scientific Survey of Meteorological Sensitive Diseases in China’s Regional Population,” No. 2017FY101200): https://www.ncmi.cn/phda/dataDetails.do?id=CSTR:17970.11.A0006.202109.581.V1.0-V1.0; Automatic Urban and Rural Network (AURN): https://uk-air.defra.gov.uk/networks/network-info?view=aurn; UK National Statistics (ONS): https://www.ons.gov.uk/peoplepopulationandcommunity/birthsdeathsandmarriages/deaths/methodologies/userguidetomortalitystatisticsjuly2017.
