# Supplementary material for: Development and two-city validation of a Temperature-Sensitive Air Quality Health Index incorporating synergistic effects of air pollution and thermal stress
Source: Front Public Health. 2026 Aug 26;14:1806127. doi: 10.3389/fpubh.2026.1806127 (PMC13556664; doi:10.3389/fpubh.2026.1806127)
Supplement: Supplementary file 1 [file Data_Sheet_1.docx]

Supplementary Table S1. Summary of data sources and characteristics for Ningbo and London.

| Item | Ningbo, China | London, UK |
| --- | --- | --- |
| Study period | 2014–2017 (full year) | 2014 – 30 June 2017 |
| Mortality | All‑cause daily death counts (total, age‑stratified, gender‑stratified) | All‑cause daily death counts for adults (≥18 years) |
| Mortality source | National Science and Technology Basic Resources Survey Program | Office for National Statistics (ONS) registry |
| Air pollutants | NO₂, SO₂, CO, O₃, PM₁₀, PM₂.₅ (daily concentrations) | NO₂, SO₂, CO, O₃, PM₁₀, PM₂.₅ (daily concentrations) |
| Meteorological variables | Daily mean temperature, daily maximum temperature | Daily mean temperature, daily maximum temperature |
| Data source for pollution & weather | Multiple city‑wide monitoring stations (aggregated) | Three AURN stations: Bloomsbury, N. Kensington, Camden Kerbside |
| Spatial resolution | City‑level (aggregated) | City‑level (mean of three stations) |
| Temporal resolution | Daily | Daily |

Supplementary Table S2. Model fit statistics for distributed lag models of air pollutants and meteorology factors associated with daily deaths (lag 0–5 days) in Ningbo, 2014-2017.

| Model(Ningbo) | AIC | BIC |
| --- | --- | --- |
| P + T + H y (lag0) | 7442.83 | 7461.80 |
| P + T + H (lagl) | 7335.65 | 7354.62 |
| P + T + H (lag2) | **7210.70** | **7229.68** |
| P + T + H (lag3) | 7336.42 | 7355.40 |
| P + T + H (lag4) | 7275.79 | 7294.76 |
| P + T + H (lag5) | 7211.23 | 7230.20 |
| P + T + H (lag2) | 11428.74 | 11628.77 |
| P + T + H + P * T (lag2) | 11430.49 | 11635.79 |
| P + T + H + P * H (lag2) | 11430.44 | 11635.74 |
| P + T + H + T * H (lag2) | **11427.87** | **11633.17** |
| P + T + H + P * T + H * T (lag2) | 11429.79 | 11640.35 |
| P + T + H + P * T + P * H (lag2) | 11432.31 | 11642.87 |
| P + T + H + P * H + T * H (lag2) | 11429.87 | 11640.43 |
| P + T + H + P * T + P * H + T * H (lag2) | 11431.78 | 11647.61 |
| P + T + H + P * T + P * H + T * H + P * T * H (lag2) | 11429.05 | 11650.14 |

*AIC = Akaike Information Criterion; BIC = Bayesian Information Criterion. All models were adjusted for long‑term and seasonal time trends using natural cubic splines of calendar time and for day‑of‑week effects.

P refers to a weighted air pollutant index, lagged by 2 days; T is the deviation of mean temperature from the 22.8℃, lagged by 2 days; R is the deviation of mean relative humidity from the 50%, lagged by 2 days.

Supplementary Table S3. The estimated weights for air pollutants in a multi-pollutant weighting model.

| Air pollutant | PM_10_ | PM_2.5_ | O_3_ | SO_2_ | CO | NO_2_ |
| --- | --- | --- | --- | --- | --- | --- |
| Weight in Ningbo ^*^ | 0.55 | 0.12 | 0.000 | 0.18 | 0.03 | 0.12 |
| Weight in London^*^ | 0.77 | 0.000 | 0.001 | 0.018 | 0.21 | 0.000 |

^*^Weights estimated for Ningbo using WQS regression. Separate weights were estimated for London using the same method (available upon request).

Supplementary Table S4. Comparative statistics of the AQHI and TS-AQHI in Ningbo and London, 2014-2017. SD, standard deviation; Min, minimum; P25, lower quartile; P50, median; P75, upper quartile; Max, maximum; IQR, interquartile range.

| Index | Mean | SD | Min | P25 | Median | P75 | Max | IQR |
| --- | --- | --- | --- | --- | --- | --- | --- | --- |
| Ningbo |  |  |  |  |  |  |  |  |
| AQHI | 2.36 | 1.27 | 0.00 | 1.48 | 2.08 | 2.88 | 10.00 | 1.41 |
| TS-AQHI | 4.40 | 1.98 | 0.00 | 3.01 | 4.20 | 5.84 | 10.00 | 2.83 |
| London |  |  |  |  |  |  |  |  |
| AQHI | 2.84 | 1.19 | 0.00 | 2.06 | 2.66 | 3.32 | 10.00 | 1.26 |
| TS-AQHI | 3.40 | 1.28 | 0.00 | 2.52 | 3.14 | 4.10 | 10.00 | 1.58 |

Supplementary Table S5. Subgroup analysis of mortality associations with TS-AQHI and AQHI by age and sex in Ningbo and London, 2014–2015. *β* (95% CI), regression coefficient with 95% confidence interval; *R²*, model explanatory power (deviance explained). Age ≥ 65, daily number of deaths among individuals aged ≥ 65 years. Age < 65, daily number of deaths among individuals aged < 65 years. Female, daily number of deaths among females. Male, daily number of deaths among males.

| Level & Index | *β* (95% CI) | *R^2^* | *p*_value |
| --- | --- | --- | --- |
| Age ≥ 65 |  |  |  |
| AQHI | 0.05 (0.04, 0.06) | 20.5% | <0.001 |
| TS-AQHI | 0.04 (0.04, 0.05) | 25.1% | <0.001 |
| Age < 65 |  |  |  |
| AQHI | 0.01 (0.00, 0.02) | 7.5% | 0.023 |
| TS-AQHI | 0.01 (0.01, 0.02) | 8.2% | <0.001 |
| Female |  |  |  |
| AQHI | 0.05 (0.04, 0.06) | 16.0% | <0.001 |
| TS-AQHI | 0.04 (0.03, 0.05) | 20.3% | <0.001 |
| Male |  |  |  |
| AQHI | 0.04 (0.03, 0.05) | 15.6% | <0.001 |
| TS-AQHI | 0.03 (0.03, 0.04) | 18.9% | <0.001 |

Supplementary Equation S1

$$\text{}$$

Where:

- The form $\sum_{\text{k=1}}^{\text{p}} \text{w}_{\text{t}}\text{ }\text{X}_{\text{kt}}$is interpreted as the air pollutant mixture; W_k_ is the relative weight of pollutant k in the air pollutant mixture; X_kt_ is the concentration of pollutant k on day t.
- β is the effect coefficient of air pollutant mixture on the given health outcome.
- The variable, confounders_t_ represents other time-varying variables related to the health outcome such as humidity and weekend.

Supplementary Equation S2

The AQHI was then modeled based on this exposure-response relationship as follows:

$$\text{E}\text{R}_{\text{t}}\text{ }\text{=}\text{ }\text{100*}\left[ \exp\left( \text{β}\sum_{\text{k}\text{=1}}^{\text{p}} \text{w}_{\text{k}}\text{X}_{\text{kt}} \right)\text{ }\text{-}\text{ }\text{1} \right]$$

$$\text{AQHI}\text{ }\text{=}\text{ }\text{10×}\text{E}\text{R}_{\text{kt}}\text{/}\text{E}\text{R}_{\text{kmax}}$$

Where:

- $\text{β}\sum_{\text{k=1}}^{\text{p}} W_{\text{k}}\text{X}_{\text{kt}}\text{ }$represents the air pollutant mixture, with $W_{\text{k}}$ denoting the relative weight of pollutant k in the mixture and $\text{X}_{\text{kt}}$ representing the concentration of pollutant k on day t.
- β is the coefficient of the indicator variable estimated by the GAM, which is the deaths associated with a unit increase in major component pollutants.
- ER_kt_ is the excess disease risk for different major component pollutants.
- ER_ktmax_ is the maximum value of the excess disease risk.

Supplementary Equation S3

The TS-AQHI was then modeled based on the generalized additive model as follows:

$$\text{E}\text{R}_{\text{t}}\text{ }\text{=}\text{ }\text{100*}\left[ \exp\left( g(X_{i} \right) - g(X_{0}))\text{-}\text{ }\text{1} \right]$$

$$\text{ }\text{TS-AQHI}\text{ }\text{=}\text{ }\text{10×}\frac{\text{(}\text{E}\text{R}_{\text{kt}}\text{ }\text{- }\text{E}\text{R}_{\text{k}\text{min}})}{\max(E\text{R}_{\text{kt}} - E\text{R}_{\text{k}\text{min}})}$$

Where:

- $g(.)$denotes the predicted value on the link scale (log) from the GAM.
- $X_{i}$ is the vector of observed exposures on day *i* (air pollutant mixture, temperature deviation, relative humidity), and $X_{0}$is the reference exposure vector. The reference scenario was defined as: air pollutant mixture = 0; temperature deviation = 0 °C; relative humidity deviation = 0 %.
- ER_kt_ is the excess disease risk for different major component pollutants.
- ER_ktmin_ is the minimum value of the excess disease risk.

Supplementary Figure S1. Location of the study areas and monitoring stations. Regional map showing the geographical positions of Ningbo, China (approximately 30°N, 121°E) and London, UK (approximately 51°N, 0°W). Note: The three AURN stations are located in central London and do not represent the full geographic extent of the city, which are used here as representative city-wide monitoring sites.

Supplementary Figure S2. Trends in Air Pollution, Heat, and Mortality in Ningbo and London, 2014-2017. (a) Weekly mean temperature (°C) over the year, stratified by city and year. (b) Annual mean CO concentration (mg/m^3^) trend. (c) Annual mean NO_2_ concentration ( μg/m^3^) trend. (d) Annual mean O_3_ concentration ( μg/m^3^) trend. (e) Annual mean PM_10_ concentration (μg/m^3^) trend. (f). Annual mean PM_2.5_ concentration ( μg/m^3^) trend. (g) Annual mean SO_2_ concentration ( μg/m^3^) trend. Note: All pollutant trends are presented as annual means, with cities differentiated by color and line style.
